# Gene expression profile of human colorectal cancer identified NKTR as a biomarker for liver metastasis

**DOI:** 10.18632/aging.204242

**Published:** 2022-08-23

**Authors:** Rui Bai, Zhong Shi, Dan Li, Donger Zhou, Wei-Ting Ge, Shu Zheng

**Affiliations:** 1 Cancer Institute, Key Laboratory of Cancer Prevention and Intervention, China National Ministry of Education, The Second Affiliated Hospital, School of Medicine, Zhejiang University, Hangzhou 310002, Zhejiang, China; 2 Department of Medical Oncology, Key Laboratory of Cancer Prevention and Intervention, Ministry of Education, The Second Affiliated Hospital, Zhejiang University School of Medicine, Hangzhou 310009, Zhejiang, China; 3 Cancer Center, Zhejiang University, Hangzhou 310058, Zhejiang, China; 4 Department of Medical Oncology, Institute of Cancer and Basic (Medicine ICBM), Chinese Academy of Sciences, Cancer Hospital of the University of Chinese Academy of Sciences, Hangzhou 310022, Zhejiang, China; 5 Department of Surgery, The Second Affiliated Hospital, Zhejiang University School of Medicine, Hangzhou, Zhejiang, China

**Keywords:** colorectal cancer, liver metastasis, NKTR, proliferation, migration and invasion

## Abstract

Objective: Liver metastasis is one of the prognostic factors of colorectal cancer (CRC). The aim of this study is to identify biomarkers that facilitate easier detection of liver metastasis.

Methods: Significance Analysis of Microarrays (SAM) and Array Data Analyzer (ADA) were applied used for the analysis of differentially differently expressed mRNAs. mRNA expression was verified by quantitative real-timer reverse transcriptiontase polymerase chain reaction (qRT-PCR). Immunohistochemistry were was used to show natural killer-tumor recognition (NKTR) expression in CRC. NKTR-knockdown CRC cells were constructed obtained by using short hairpin RNA (shRNA). Followed by CCK-8 assay, plate colony formation test, and transwell assay were used to evaluate the influence of NKTR on cell proliferation, migration, and invasion *in vitro*.

Results: SAM yielded showed 256 up-regulated and 224 down-regulated differentially differently expressed genes. Seven genes were identified by using ADA, tools and four genes were verified by using qRT-PCR. Three genes (metastasis associated lung adenocarcinoma transcript 1 (MALAT1), nuclear factor I/B (NKTR), and nuclear factor I/B (NFIB)) showed a statistically significant considerabley difference between CRC with and liver metastasis and CRC without liver metastasis. Immunohistochemical analysis showed that NKTR expression was much lower in primary CRC with liver metastasis than that in primary CRC without liver metastasis. The NKTR protein plays a role in the lytic function of natural killer (NK) cells and it has been rarely studied in the CRC. The down-regulation of NKTR by shRNA interference in CRC cells increased cell proliferation, migration, and invasion *in vitro*.

## INTRODUCTION

Colorectal cancer (CRC) is the third most common cancer in men and the second most common cancer in women. CRC is also the fourth most frequently diagnosed cancer and the second leading cause of cancer death in the United States. There were an estimated 101,420 new cases of colon cancer and 44,180 new cases of rectal cancer in 2019 [1].

The general 5-year survival rate of CRC is estimated to be 65%, but it declines to 11% if distant metastases are present [2]. Approximately 20–25% of patients with CRC present synchronous metastasis, and nearly 50% of patients with CRC develop liver metastases [3]. The liver is the most common site of distant metastasis in CRC. The survival of CRC patients with liver metastasis is not determined by the status of the primary tumor but is related to the progression of the liver disease [4]. Liver metastasis is the most important prognostic factor for CRC. CRC patients with untreated liver metastasis have a poor prognosis, and the median survival time of the patients is only 6.9 months [5]. The early detection of liver metastasis is important to improve CRC survival. The identification of high-risk patients for liver metastasis can reduce its development by allowing intensive adjuvant chemotherapy or curative surgical resection. Therefore, biomarkers that facilitate the detection of liver metastasis can be useful.

CRC metastasis is a complex process involving epithelial-mesenchymal transition, migration, invasion, intravasation, survival in the bloodstream, extravasation, and final seeding and expansion in a new environment [6]. Previous studies have shown that the development of CRC, like other cancers, is a result of multiple genetic alterations. Multiple genes are involved in these processes and have been reviewed [7]. Understanding the molecular and cellular mechanisms underlying CRC formation, particularly the progression of liver metastasis, is of utmost importance.

Microarray studies can serve two distinct purposes: (a) Identify all significantly differentially expressed genes between two groups to gain insight into biological processes that differentiate the groups using gene ontology analysis or pathway analysis tools [8]; (b) Identify a parsimonious set of genes that yield a predictive model with good performance in independent cases. Several studies have used microarrays to predict CRC liver metastasis. Gene expression studies have suggested a model in which the ability to metastasize is an early event that can already be distinguished in primary tumor tissues [9].

In the present study, we have been using primary tumor tissues of CRC, including CRC with and without liver metastasis. Gene expression profiles were determined using Affymetrix Human U133 Plus 2.0 GeneChip, and CRC with and without liver metastasis were compared. Significance Analysis of Microarrays (SAM) was used to identify significant differences in the expression of genes comparing CRC liver metastasis. A novel tool, the Array Data Analyzer (ADA) finding the optimal tradeoff between fold change and *t*-test p-values was used to detect highly predictive genes. Quantitative reverse transcription polymerase chain reaction analysis (qRT-PCR) was used to verify the highly predictive genes identified using ADA and SAM. We found that natural killer-tumor recognition (NKTR) molecule was a biomarker of CRC liver metastasis.

## MATERIALS AND METHODS

### Patient samples

Primary tumor samples of CRC patients were collected for gene expression profiling analysis (19 samples with liver metastasis and 49 samples without liver metastasis) and NKTR IHC (29 samples with liver metastasis and 42 samples without liver metastasis). Patients underwent tumor resection in the Second Affiliated Hospital of Zhejiang University from 2001 to 2007. Written informed consent was provided by patients. The tumor samples used for gene expression profiling were stored at −80° C. The tumor samples used for NKTR IHC were paraffin-embedded. We identified liver metastases using computerized tomography (CT) scans. Patient and tumor characteristics are shown in Tables 1, and 2.

**Table 1:** Patient and tumor characteristics of gene expression profiling analysis.

**Characteristic**		**LM (n=19)**	**LM-N (n=49)**	**P-value**
Age	Mean(SD)	56.3(16.4)	61.1(13.7)	0.26
	Range (years)	19-78	26-91	
Gender	Male	11	30	0.80
	Female	8	19	
T classification	T1,2	2	5	0.69
	T3,4	17	44	
Lymph-node	None	4	21	0.09
	>0	15	28	
Site	Colon	10	30	0.52
	Rectum	9	19	
Histological type	Well/ Moderate	11	36	0.21
	Poor/Others	8	13	

LM: CRC with liver metastasis.

LM-N: CRC without liver metastasis.

**Table 2:** Patient and tumor characteristics of immunohistochemistry.

**Characteristic**		**LM (n=29)**	**LM-N (n=42)**	**P-value**
Age	Mean(SD)	56.7(14.3)	61.7(12.9)	0.14
	Range (years)	30-82	32-86	
Gender	Male	20	26	0.54
	Female	9	16	
T classification	T1,2	4	5	0.90
	T3,4	25	37	
Lymph node	None	7	18	0.10
	>0	22	24	
Site	Colon	15	24	0.65
	Rectum	14	18	
Histological type	Well/ Moderate	23	34	0.86
	Poor /Others	6	8	

LM: CRC with liver metastasis.

LM-N: CRC without liver metastasis.

### RNA isolation

Total RNA was isolated from frozen samples using the nuclear spin RNA II kit (Macherey-Nagel, Allentown, GA, USA). Bioanalyzer instrument (Agilent, Santa Clara, CA, USA) was used to verify the quality of the total RNA.

### Microarray expression profiling

Sample preparation, labeling, and hybridization to Affymetrix Human U133 Plus 2.0 GeneChips (Affymetrix,Santa Clara, CA, USA) were performed according to the manufacturer’s protocols. The probe hybridized with the RNA samples to obtain the expression intensity signal of each gene in each sample, which is scanned by Affymetrix scanner, and the data was read and processed by GeneChip® Operating Software Version 1.4 software. A robust multi-array average (RMA) standardization method was used to analyze the Affymetrix raw data file [cell intensity (CEL) file] [10].

### Microarray data processing

### SAM

Differentially expressed genes (DEGs) were analyzed using Significance Analysis of Microarrays (SAM) (http://www-stat.stanford.edu/;tibs/SAM/) [11].

### Hierarchical clustering

Hierarchical clustering analysis was performed using Cluster version 3.0 and Treeview version 1.0.13 (Stanford University).

### ADA

**Figure 1:**
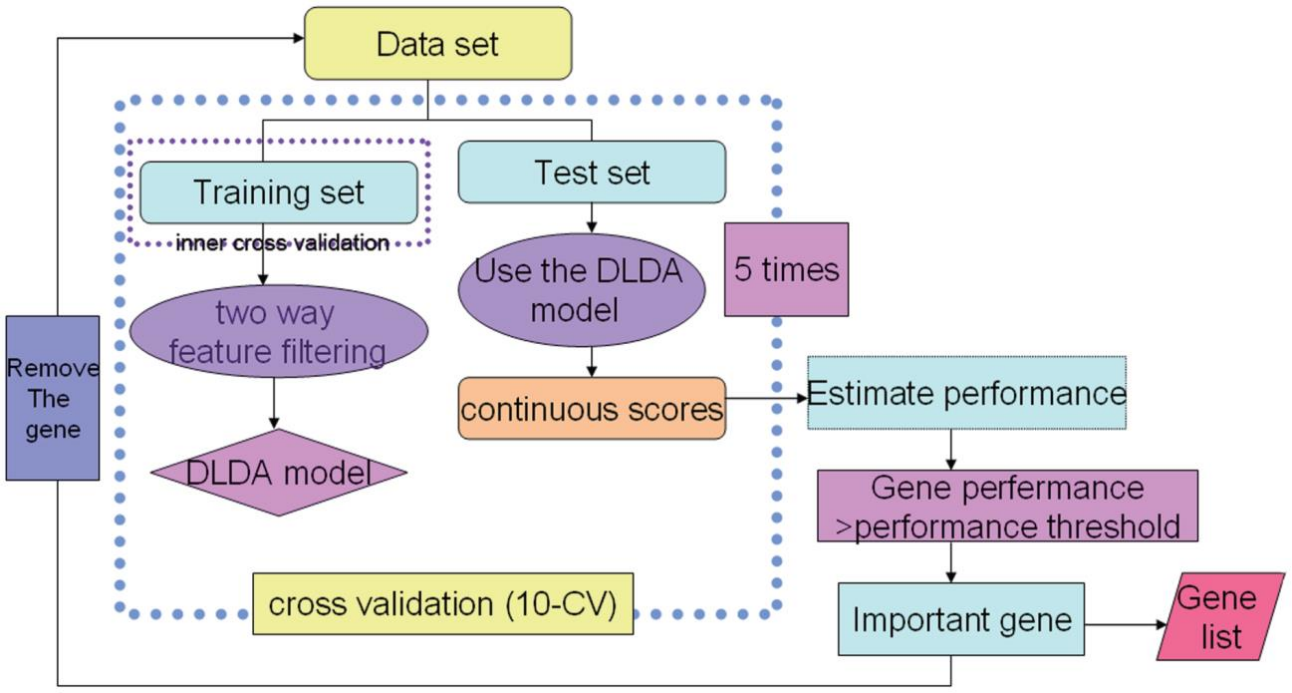
**An overview of the ADA method.** 1. “FindGeneSignature” used a grid search procedure that searched through various tradeoffs of the mean difference test and t-test. 2. “EstimatePerformance” was used to estimate model performance (ROC curve). 3. “FindImportantGenes” is a wrapper procedure that iteratively collects generated gene signatures and removes these genes from further runs.

ADA was used for various GSK drug discovery projects. A schematic overview of the ADA method is presented in Figure 1 [12].

### qRT-PCR

Differentially expressed genes were validated using qRT-PCR (Applied Biosystems, Foster City, CA, USA). Total RNA (5 μg) was reverse transcribed with RT-PCR Kit (Promega, Madison, WI, USA). 500 ng cDNA was used for qRT-PCR. The qRT-PCR primers and probes are listed in Supplementary Table 1. qRT- PCR was performed in 25 μL system with TaqMan Master Mix Regent Kit (Applied Biosystems). Human β-actin was used as internal reference control. Relative mRNA expression was calculated by the equation 2^-ΔΔCT^.

### Cell culture

Cell lines (SW480, SW620, LOVO, RKO, HT29, DLD1, and HCT116) were purchased from the American Type Culture Collection (Manassas, VA, USA) and cultured under recommended conditions.

### Immunohistochemistry (IHC) analysis

4-μm sections were cut from paraffin-embedded tissue blocks. Rat anti-human NKTR polyclonal antibody (HPA022120, Sigma-Aldrich, St. Louis, MO, USA) was used for IHC. We incubate the primary antibody at a dilution of 1:200 and incubated it overnight at 4° C. The IHC kit included a ready to use quick IHC MaxVision Kit (kit-5010, Maixin Bio, China) and DAB chromogenic Kit (DAB-0031, Maixin Bio, China). The IHC results were assessed by a semi-quantitative system as previously described [13] The positive range was 0-4: 0 (< 10%), 1 (10% - 25%), 2 (25% - 50%), 3 (50% - 75%), 4 (> 75%); the staining depth was divided into 0-3 grades: 0 was negative, 1 was light yellow, 2 was brown yellow, 3 was dark brown. The results were divided into 0-3 grades: 0 (0-1), 1 (2-3), 2 (4-5), 3 (6-7).

### NKTR knockdown using small hairpin RNA (shRNA)

shRNA lentiviral particles were used for *NKTR* knockdown (sc-78500-v, Santa Cruz, CA, USA) and mock knockdown (sc-108080, Santa Cruz, CA, USA). DLD1 cells were infected with shRNA lentiviral particles in the presence of 4 μg/mL polybrene. Puromycin (2 μg/mL) was used for screening cell lines, and NKTR expression was evaluated using western blot.

### Cell proliferation assay

Cell Counting Kit-8 (CCK-8, Shenggong, China) was used for cell proliferation assay. Cells (2×10^3^/well) were cultured in triplicate in 96-well plates. Assays were performed every day for 5 days. Briefly,10μl CCK-8 was added to each well, and then the plates were incubated at 37° C for 1 h and optical density (OD) was measured at 450 nm.

### Plate colony formation assay

Plate colony formation assay was used to measure cell colony formation rate. After digesting by trypsin, the logarithmic phase cells were blown into a single cell suspension and counted. Cells (n = 1,000) were added to each well of a 6-well plate. Plates were incubated at 37° C for 14 days, fixed with 4% paraformaldehyde, and stained with crystal violet. Viable colonies containing at least 50 cells were counted.

### Migration and invasion assays

### Migration assays

Cells (1×10^5^/well) suspended in 200 μL FBS-free RPMI-1640 were dispensed into the upper chambers of transwell (8-μm pore size). Then, 600 μL of 10% FBS RPMI-1640 was dispensed in the lower chambers. Cells migrating to the lower membrane surface were fixed with 4% paraformaldehyde, stained with crystal violet, and counted under a microscope. Six high-power microscopic fields (400×) per filter were photographed, and the numbers of cells were counted.

### Invasion assays

Matrigel was used to coat transwell chambers. The procedures of invasion assays were similar to those of the cell migration assay.

### Western blot

Cells were harvested and prepared using the Radio Immunoprecipitation Assay (RIPA) lysis buffer (Boster, China). Protein concentrations of the samples were determined using the bicinchoninic acid (BCA) protein assay (Millipore, Bedford, MA, USA). Each sample (30 μg) was boiled to denature the protein, and western blot analysis was performed. The primary antibodies used were polyclonal antibodies against NKTR (1:1,000, AAA515N, Invitrogen, Carlsbad, CA, USA) and β-actin (1:5,000, M1210-2, HuaBio, China).

### Statistical analysis

The *t*-test was used to compare continuous measurements. Chi-squared test or calibration chi-square test was used to compare categorical measurements. The nonparametric Mann-Whitney U test was used to analyze the NKTR IHC results. Significant differences were considered if the p-value was < 0.05. Data are expressed as the mean ± s.e.m. The differences among groups were determined by Student’s *t*-test using GraphPad Prism software version 8.0 (San Diego, CA, USA).

## RESULTS

### Microarray data based on SAM analysis

Gene expression profiling of 68 CRC by Affymetrix Human U133 Plus 2.0 GeneChips yielded 256 up-regulated and 224 down-regulated differentially expressed genes using SAM (fold change > 1.5 or < 0.67, p < 0.05 and FDR > 0.05) when comparing CRC without liver metastasis to CRC with liver metastasis (Supplementary Table 2).

**Figure 2:**
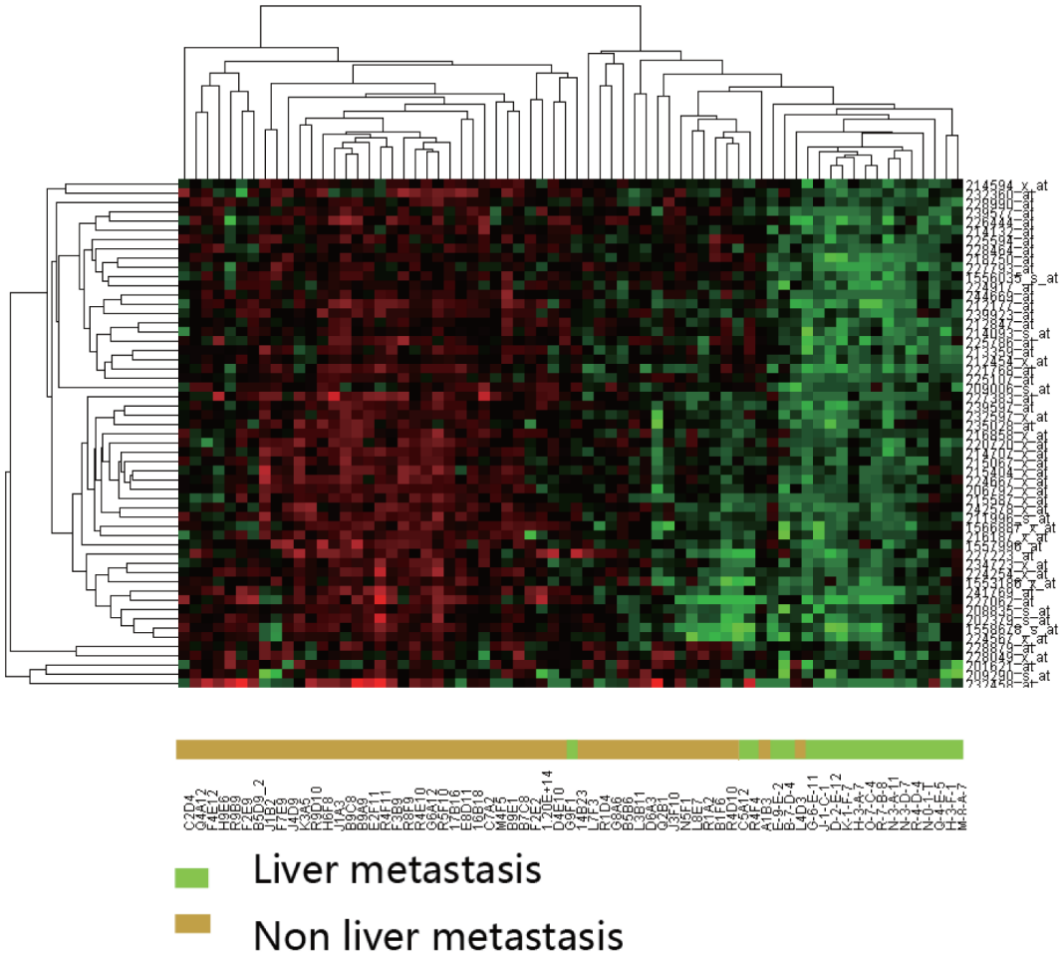
**Hierarchical clustering analysis of differentially expressed genes between CRC with and without liver metastasis.** Clustering was performed using the Pearson correlation with average linkages. Expression intensities are shown after gene and sample normalization.

DEGs were evaluated by unsupervised clustering. Samples were generally separated into two main branches: CRC with liver metastasis and CRC without liver metastasis (Figure 2).

### Data mining based on ADA

To concentrate gene markers, we used ADA. The identification of genes was combined with a 5-times 10-fold cross-validation, yielding a validated set of classificatory genes.

### qRT-PCR analysis

**Figure 3:**
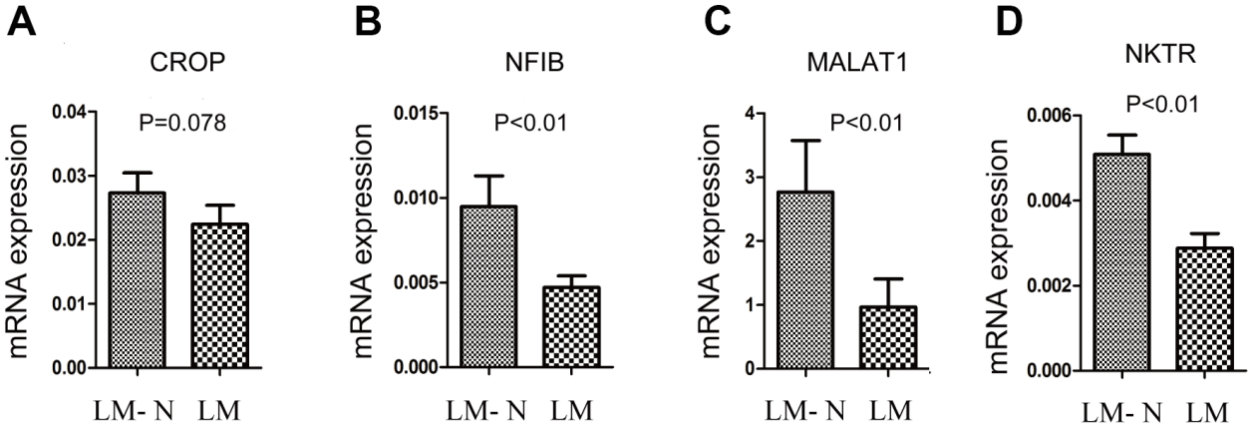
**Quantitative RT-PCR gene expression analysis of four genes.** The relative expression was calculated using the equation relative quantification (RQ) = 2^−ΔΔCT^, and β-actin was used as an internal control. Error bars indicate s.e.m. LM: CRC with liver metastasis, LM-N: CRC without liver metastasis. (**A**) CRC samples with liver metastasis had *a lo*wer mRNA expression of CROP compared to that of the CRC without liver metastasis, but p=0.078 (**B**) CRC samples with liver metastasis had *a lo*wer mRNA expression of NFIB compared to that of the CRC without liver metastasis (p<0.01). (**C**) CRC samples with liver metastasis had *a lo*wer mRNA expression of MALAT1 compared to that of the CRC without liver metastasis (p<0.01). (**D**) CRC samples with liver metastasis had *a lo*wer mRNA expression of NKTR compared to that of the CRC without liver metastasis (p<0.01).

Among eight discriminating probes, seven genes could be identified by using UniGene: *MALAT1*, *HNRPDL*, *HNRPA2B1*, *SFPQ*, *NKTR*, *CROP* and *NFIB*. (Supplementary Table 3) Four genes (*MALAT1*, *NKTR*, *CROP* and *NFIB*) were also in the list from detected using the SAM analysis, and qRT-PCR was performed to validate gene expression. CRC samples with liver metastasis had *a lo*wer mRNA expression of *NKTR*, *NFIB*, and *MALAT1* compared to that of the CRC without liver metastasis (P < 0.01) (Figure 3).

### IHC analysis of NKTR expression

**Figure 4:**
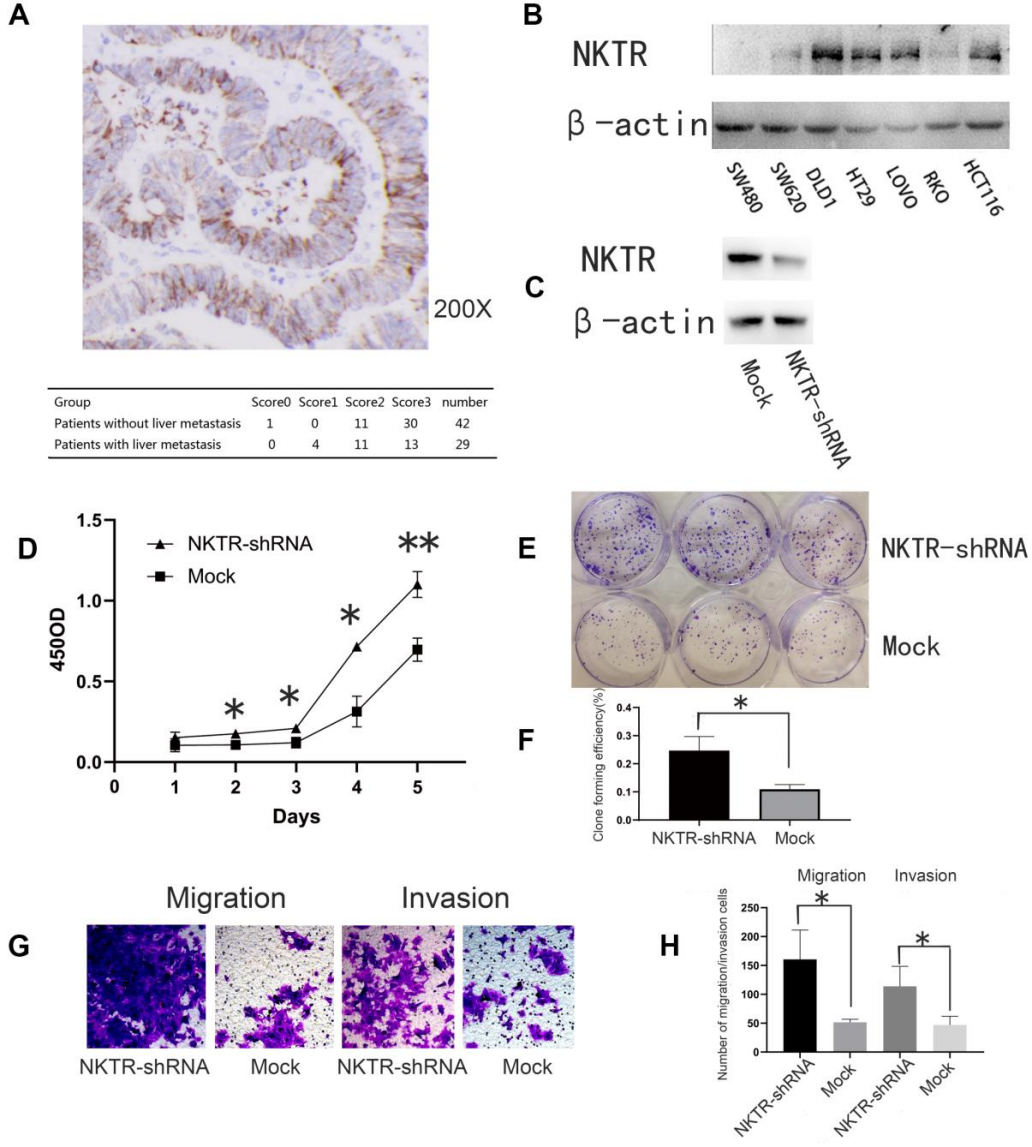
(**A**) Immunohistochemical staining and assessment of NKTR in primary CRC tissue. (**B**) Western blot showed NKTR expression in seven CRC cell lines. β-actin was used as an internal control. (**C**) Western blot showed NKTR expression in DLD1-NKTR-shRNA and mock. (**D**) Effect of NKTR expression of DLD1 cell proliferation. Error bars indicate s.e.m., n = 3. *P < 0.05, **P < 0.01 (Student’s *t*-test). (**E**, **F**) Effect of NKTR expression on DLD1 cell colony formation. Error bars indicate s.e.m., n = 3. **P < 0.05 (Student’s *t*-test). (**G**, **H**) Effect of NKTR expression on DLD1 cell migration and invasion. Data are representative of each group or expressed as the mean ± s.e.m. of cells per six high power fields. *P < 0.05 (Student’s *t*-test).

We showed that NKTR expression occurred in CRC cells and was much lower in CRC with liver metastasis than that in the CRC without liver metastasis (P = 0.017, nonparametric Mann-Whitney U test table) (Figure 4A).

### Roles of NKTR in CRC cell growth, migration, and invasion

Western blot was used to quantify NKTR expression in seven human CRC cells (Figure 4B). NKTR was most highly expressed in DLD1 cells. Western blot verified the knockout efficiency of NKTR (Figure 4C).

Compared to the mock group, the knockdown of *NKTR* significantly increased the proliferative capacity of the NKTR-shRNA group (P < 0.01 *t*-test) (Figure 4D). In addition, compared to the mock group, the colony formation of the NKTR-shRNA group significantly increased (P < 0.05, *t*-test) (Figure 4E, 4F). Compared to the mock group, the migration and invasion of the NKTR-shRNA group increased significantly (P < 0.05, *t*-test) (Figure 4G, 4H). We have verified the results in Figure 4 in one other CRC cell line Supplementary Figure 1.

## DISCUSSION

Cancer metastasis has been extensively studied and characterized as a complex, multistep process. Cancer cells acquire the metastatic phenotype by accumulating genetic or epigenetic alterations [8]. These genetic or epigenetic alterations are already present in the primary tumor, based on evidence that the gene expression signatures of the primary tumor predict metastasis in patients with breast cancer. In our study, primary tumor tissues of 68 CRC patients (with or without liver metastasis) were collected to determine gene expression profiles. qRT-PCR was used to verify the microarray data. We showed that *MALAT1*, *NFIB*, and *NKTR* had different expression levels when comparing CRC with and without liver metastasis. IHC analysis and *in vitro* analysis showed that NKTR was a biomarker of CRC liver metastasis.

Gene expression studies suggested a model in which the ability to metastasize was an early event that could already be distinguished in primary tumors. The potential for metastasis is already encoded in the primary tumor and is detectable by gene expression. The prediction of CRC liver metastasis can be diagnosed with primary CRC tissue.

Traditional SAM analysis cannot concentrate markers. To overcome this limitation, we used ADA, which could discover highly predictive genes. Cancer is a heterogeneous disease, and different types (normal distribution and non-normal distribution) of true biomarkers coexist in the same data set. Non-outlier and outlier detection statistics are used to identify normally distributed and non-normally distributed biomarkers separately. The ADA was developed to discover biomarkers by detecting several effective tradeoffs between two statistics (non-outlier and outlier detection statistics). ADA can adapt to the dataset by finding a good balance of the two statistics based on the performance of the cross-validated model. We obtained seven genes (*MALAT1*, *HNRPDL*, *HNRPA2B1*, *SFPQ*, *NKTR*, *CROP* and *NFIB*). Four of them (*MALAT1*, *NKTR*, *CROP* and *NFIB*) were also detected using SAM, which was also based on the fold change and *t*-test p-value. However, without a good balance of the two statistics and cross-validation, SAM analysis could not concentrate highly predictive genes.

After qRT-PCR validation, we found that the gene expression of *MALAT1*, *NKTR* and *NFIB* was significantly different in CRC with and without liver metastasis (P < 0.01). Since the function of NKTR in CRC has not been studied, we investigated the function of NKTR in CRC. The NKTR sequence encodes a membrane-anchored protein with a hydrophobic amino terminal domain and a cyclophilin-like PPIase domain. The NKTR protein plays a role in the lytic function of natural killer (NK) cells [14, 15]. Its expression is increased by IL2 activation of the cells [16]. Interference with normal NKTR expression in HL-60 cells markedly suppresses many differentiation-associated events [17]. Overexpression of the centrosome-associated serine/threonine kinase Aurora Kinase A (AURKA) has been demonstrated in advanced prostate cancer and high-grade prostatic intraepithelial neoplasia lesions. The NKTR variant differentiates between AURKA Phe31/Phe31 and Ile31/Ile31 prostate RNA [18]. The expression of NKTR was lower in CRC with liver metastasis than that in the CRC without liver metastasis. There have been few reports on NKTR in CRC, but, in 2008, Pantaleo [19] found that the expression of NKTR in primary CRC with simultaneous liver metastases was lower than that in primary CRC with metachronous liver metastases. In addition, shRNA knocking out *NKTR* expression can significantly promote tumor cell migration and invasion, suggesting that NKTR may play an inhibitory role in tumor metastasis. The low expression of NKTR in primary CRC may be an important marker for predicting liver metastasis. According to current literature reports, NKTR is closely related to NK cell immunity. On one hand, liver metastasis may be a process closely related to immunity. On the other hand, genes that have been studied most frequently in immunity may also play a very important role in CRC liver metastasis. For the first time, our study showed that NKTR was a negative regulator of the progression and metastasis of CRC.

## CONCLUSIONS

ADA can concentrate target genes. NKTR is an important marker in CRC liver metastasis. CRC patients may benefit from the accurate evaluation and realistic treatment strategies for their disease with the help of this potential prediction marker.

## Supplementary Material

Supplementary Figure 1

Supplementary Table 1

Supplementary Table 2

Supplementary Table 3
