# Supplementary material for: Gene expression profile of human colorectal cancer identified NKTR as a biomarker for liver metastasis
Source: Aging (Albany NY). 2022 Aug 23;14(16):6656–67. doi: 10.18632/aging.204242 (PMC9467399; doi:10.18632/aging.204242)
Supplement: Supplementary Figure 1 [file aging-14-204242-s001.pdf]

SUPPLEMENTARY FIGURE

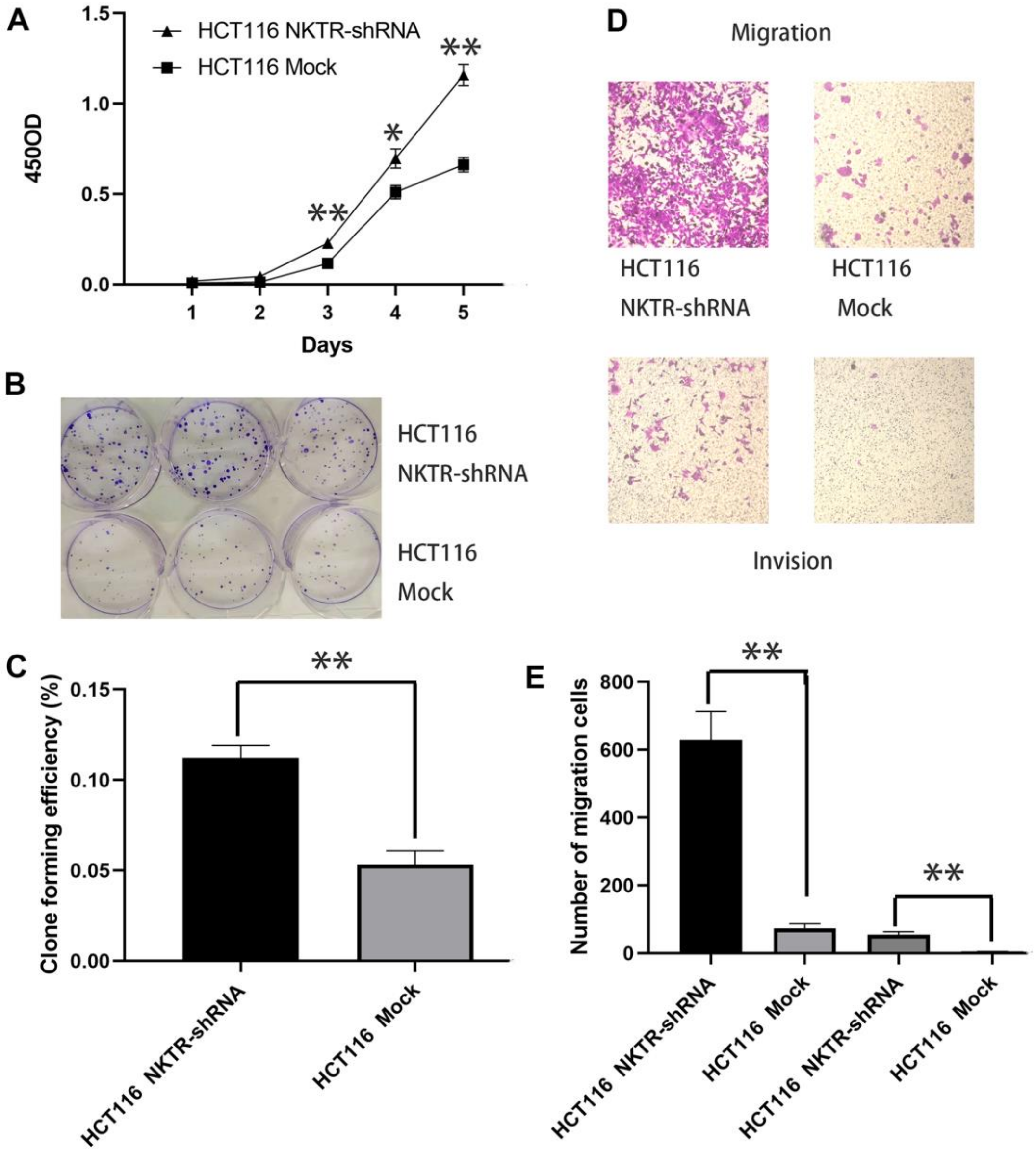

Supplementary Figure 1. (**A**) Effect of NKTR expression of HCT116 cell proliferation. Error bars indicate s.e.m., n = 3. *P < 0.05, **P < 0.01 (Student's *t*-test). (**B**, **C**) Effect of NKTR expression on HCT116 cell colony formation. Error bars indicate s.e.m., n = 3. **P < 0.05 (Student's *t*-test). (**D**, **E**) Effect of NKTR expression on HCT116 cell migration and invasion. Data are representative of each group or expressed as the mean ± s.e.m. of cells per six high power fields. *P < 0.05 (Student's *t*-test).
