# Supplementary material for: Gene expression profile of human colorectal cancer identified NKTR as a biomarker for liver metastasis
Source: Aging (Albany NY). 2022 Aug 23;14(16):6656–67. doi: 10.18632/aging.204242 (PMC9467399; doi:10.18632/aging.204242)
Supplement: Supplementary Table 1 [file aging-14-204242-s002.pdf]

Supplementary Table 1. The qRT-PCR primers and probes.

Gene symbol	Forward primer	Pober	Reverse primer
CROP	5'-CAACGTCGACA ATTGAAAGC-3'	5'-FAM-ACCCGGGACTG GGCATCTCC-TAMRA-3'	5'-GCATAGCCCAT GTGTTGTTT-3'
NFIB	5'-CTTATGACCCAT CCAGTCCA-3'	5'- FAM-CCAGCCAGTCCTGGT ACCTGGG- TAMRA-3'	5'-TGACACTTGGA AAGGAACCA-3'
NKTR	5'-TGCAGAACCTG AACCGAAGAT-3'	5'-FAM-CCTGATGTTGCA CCCATTGTAAGTGATCA G-TAMRA-3'	5'-CGTCCAGACTT TGATACAGATGGT-3'
MALAT1	5'-GGAAGTAATTCA AGATCAAGA-3'	5'-FAM-CCTGTCTGCTGC TGTCTTCCTA-TAMRA-3'	5'-CAGTCCTAGCT TCATCAA-3'
β-ACTIN	5'-GGTCATCACCAT TGGCAATG-3'	5' -FAM-CGGTTCCGCTGCCCT GAGGC-TAMRA-3'	5'-TCCATGCCCAG GAAGGAA-3'

FAM, 6-carboxyfluorescein; TAMRA, 6-carboxytetramethylrhodamine.
