# Supplementary material for: Gene expression profile of human colorectal cancer identified NKTR as a biomarker for liver metastasis
Source: Aging (Albany NY). 2022 Aug 23;14(16):6656–67. doi: 10.18632/aging.204242 (PMC9467399; doi:10.18632/aging.204242)
Supplement: Supplementary Table 3 [file aging-14-204242-s004.pdf]

Supplementary Table 3. Eight discriminating probes (seven genes).

225107_at	HNRPA2B1	heterogeneous nuclear ribonucleoprotein A2/B1	HNRNP, HNRNPA2, HNRNPB1, HNRPA2, HNRPA2B1, HNRPB1, RNPA2, SNRPB1	Heterogeneous nuclear ribonucleoproteins A2/B1, hnRNP A2 / hnRNP B1
212454_x_at	HNRPDL	heterogeneous nuclear ribonucleoprotein D-like	HNRNP, HNRPDL, JKTBP, JKTBP2, laAUF1	
221768_at	SFPQ	splicing factor proline/glutamine rich (polypyrimidine tract binding protein associated)	POMP100, PSF, SFPQ	100 kDa DNA-pairing protein, DNA-binding p52/p100 complex, 100 kDa subunit, PSF, PTB-associated-splicing factor, Polypyrimidine tract-binding protein-associated-splicing factor, Splicing factor, proline- and glutamine-rich, hPOMp100
202379_s_at	NKTR	natural killer-tumor recognition sequence	DKFZp686F1754, DKFZp686G0426, DKFZp686J06106, DKFZp686N24126, MGC90527, NKTR, p104	NK-TR protein, NK-tumor recognition protein, Natural-killer cells cyclophilin-related protein
208835_s_at	CROP	cisplatin resistance-associated overexpressed protein	CROP, LUC7A, O48, OA48-18	CRE-associated protein 1, CREAP-1, Cisplatin resistance-associated overexpressed protein, Luc7A, Okadaic acid-inducible phosphoprotein OA48-18, cAMP regulatory element-associated protein 1
209290_s_at	NFIB	nuclear factor I/B	NFI-RED, NFIB, NFIB2, NFIB3	CCAAT-box-binding transcription factor, CTF, NF-I/B, NF1-B, NFI-B, Nuclear factor 1 B-type, Nuclear factor 1/B, TGGCA-binding protein
1558678_s_at	MALAT1	metastasis associated lung adenocarcinoma transcript 1 (non-coding RNA)	MALAT-1, MALAT1, PRO1073	
224567_x_at	MALAT1	metastasis associated lung adenocarcinoma transcript 1 (non-coding RNA)	MALAT-1, MALAT1, PRO1073	
